# Analysis and characterization of the genes encoding the Dicer and Argonaute proteins of *Schistosoma japonicum*

**DOI:** 10.1186/1756-3305-3-90

**Published:** 2010-09-17

**Authors:** Rong Luo, Xiangyang Xue, Zhangxun Wang, Jun Sun, Ying Zou, Weiqing Pan

**Affiliations:** 1 Institute for Infectious Diseases and Vaccine Development, Tongji University School of Medicine, 1239 Siping Road, Shanghai 200092, China; 2 Department of Pathogenic Biology, Second Military Medical University, 800 Xiang Yin Road, Shanghai 200433, China; 3 Department of Microbiology, Wenzhou Medical College, University-town, Wenzhou 325035, China

## Abstract

**Background:**

The Dicer and Argonaute(AGO) proteins within the small RNA regulatory pathways (SRRPs) play an indispensable role in regulation of gene expression. In this study, we analyzed two genes, Dicer and Argonaute, from *Schistosoma japonicum*, along with their expression through a combination of bioinformatics and experimental approaches.

**Results:**

Our results indicate that one Dicer and four Argonaute genes exist in *Schistosoma japonicum*, termed SjDicer and SjAGO1, 2, 3, and 4, respectively. SjDicer encodes 2590 amino acid residues that contains 5 conserved domains, including one amino-terminal helicase domain, one PAZ (Piwi-Argonaut-Zwille) domain, two RNAse III domains, and one dsRNA-binding domain. SjAGO1, 2, and 3 encode 1009, 945, and 904 amino acid residues, respectively, all of which contain PAZ and PIWI domains. In addition, we analyzed the expression profiles of SjDicer and SjAGO1 genes by qRT-PCR in eggs, miracidium, cercariae, schistosomula, and adult worms. Results showed consistent expression of both SjDicer and SjAGO1 in different stages; however, their expression levels were stage-dependent, with the highest being in the miracidium stage.

**Conclusions:**

This study provided the sequence of the Dicer and Ago genes of *S. japonicum *and their expression profiles which are essential for further investigation of functions of miRNA in *Schistosoma japonicum*.

## Background

Small RNA-mediated gene silencing pathways play important and diverse roles in development and differentiation of organisms[1-5]. Dicer and Argonaute (AGO) are the two core proteins involved in this pathway [6]. Dicer processes linear dsRNA into small interfering RNA (siRNA) duplexes and also excises mature miRNAs from pre-miRNAs in the cytoplasm [7]. Mature miRNAs and siRNAs bind to distinct members of the Argonaute protein family to form ribonucleoprotein complexes that recognize partially, or nearly perfect, complementary nucleic acid targets, and then mediate a variety of regulatory processes, including transcriptional and post-transcriptional gene silencing [8-10].

Dicer belongs to the Ribonuclease III family and has many subtypes, all of which contain one PAZ (Piwi-Argonaut-Zwille) domain, two RNAse III domains, and one RNA-binding domain (RBD) [11]. Besides the PAZ domain, all Argonaute proteins share the structural features of PIWI domains[12]. Both Dicer and Argonaute proteins are highly conserved between species, and many organisms encode multiple members of the family[13]. Although those subtypes are similar to each other in amino acids sequence and structure, they participate in different small RNA regulatory pathways (SRRPs)[6,14]. In general, in miRNA pathway Dicer-1 cleaves pre-miRNAs to form mature miRNAs, which are transferred to Ago1 for silencing of target mRNAs[3,15,16]. In siRNA pathway, long double-stranded RNA is cleaved by Dicer-2 to form mature siRNA and then complexes with a RISC-like structure for silencing of target mRNA[6].

Human schistosomiasis is one of the most prevalent and serious parasitic diseases in tropical and subtropical regions[17-19]. Schistosomiasis is mainly caused by species of *Schistosoma *including *S. japonicum*, *S. mansoni*, *and S. haematobium. *Recently, Gomes[20] and Krautz-peterson and Skelly[21] reported the discovery of Dicer and the Ago family using bioinformatics and experimental approaches in *S. mansoni*. We have previously identified small regulatory RNA in *S. japonicum*, including miRNAs, by traditional cloning and high-throughput sequencing approaches[22,23]. To further investigate potential functions of the small RNAs and their role in the regulatory networks in *S. japonicum*, it is meaningful and important to analyse and characterize the important proteins, Dicer and Ago. In this study, we obtained the sequence of the Dicer and Ago genes in *S. japonicum *through a combination of bioinformatics and experimental methods and analyzed their expression profiles in different stages of the parasite.

## Materials and methods

### Sequence retrieval of Dicer and Argonaute families

*S. japonicum *genome and transcriptome sequence data were downloaded from SCBIT (http://www.scbit.org/pages/index.do), as well as the Sanger Institute (http://www.sanger.ac.uk/) and NCBI (http://www.ncbi.nlm.nih.gov/). Amino acid sequences of *D. melanogaster *and *S. mansoni *orthologs were used as queries. The BLASTp algorithm, underpinned by the Pfam and CDD databases was used for searches of conserved protein domains or motifs.

### Multiple alignments and phylogenetic analyses

Multiple alignments of protein sequence were performed by ClustalX and phylogenetic analyses were conducted in MEGA 4[24]. Phylogenetic trees of these sequences were inferred using the Neighbor-Joining method[25]. The bootstrap consensus tree inferred from 1000 replicates was used to represent the evolutionary history of the taxa analyzed. Branches corresponding to partitions reproduced in less than 50% bootstrap replicates are collapsed. The percentage of replicate trees in which the associated taxa clustered together in the bootstrap test (1000 replicates) is shown next to the branches [26]. The tree was drawn to scale, with branch lengths in the same units as those of the evolutionary distances used to infer the phylogenetic tree. All positions containing gaps and missing data were eliminated from the dataset.

### Parasites

Parasite culturing was performed as described previously[22]. *S. japonicum *was maintained by routine passage through *Oncomelania hupensis *snails and BALB/c mice. The infected snails were induced to shed cercariae under light exposure for 2 h, and the cercariae were recovered by sedimentation on ice. Schistosomula or adult worms were obtained by liver perfusion of mice after 3 or 6 wks of infection. Eggs were obtained from liver of mice 6 wks after infected by *S. japonicum*. Eggs were then incubated to produce miracidium at room temperature and normal light conditions. After collection, all freshly isolated samples were washed three times with 1 × phosphate buffered saline (PBS), pH 7.4, and were immediately used for extraction of total RNA or stored in liquid nitrogen. All procedures performed on animals within this study were conducted in accordance with and by approval of the Tongji University Committee on Use and Care of Animals.

### PCR amplification and RACE (Rapid Amplification of cDNA Ends)

Total RNA was extracted using Trizol reagent. To make sure there was no contamination by *S. Japonicum *DNA, total RNA was treated with RNase-free DNase I. And then total RNA was quantified using a spectrophotometer, and 1 μg of RNA was reverse transcribed using an oligo-dT primer. Reverse-transcribed cDNA samples were used as templates for PCR amplification. The 5'UTR and 3'UTR of genes were amplified via RACE (rapid amplification of cDNA ends) using the Full RACE kit from TaKaRa, and target sequences were then cloned and sequenced.

### Real-time PCR analysis

Eggs, cercariae, schistosomula, and adult worms were collected for real-time PCR to quantify developmental expression of SjDicer and SjAGO. Reverse-transcribed cDNA was prepared as above-mentioned and was used as templates for real-time PCR amplification using SYBR Green (TaKaRa) and a 7300 Real-Time PCR System (Applied Biosystems). Specific primers for S. japonicum GAPDH were used as a control. Relative expression was calculated according to the 2-ΔΔCt method[27].

## Results

### Data mining of Dicer- and Argonaute-related sequences in *S. japonicum*

Homology analyses at the amino acid level revealed that the members of small RNA regulatory pathways (SRRPs) are well conserved among diverse organisms of *D. melanogaster*, *C. elegans*, *Homo sapiens*, *Mus musculus *and *S. Mansoni*[20]. Due to the closer relationship between *S. japonicum *and *S. mansoni*, we chose SmDicer and SmAGO1/2/3/4 as reference sequences to retrieve their orthologs in *S. japonicum*.

Transcriptome analysis via BLAST of *S. japonicum *revealed one EST (AY810618) of 538 bp on supercontig CCON0000000957 that exhibited sequence similarity to proteins belonging to the Dicer family. Based on data mining of the transcriptome database of *S. japonicum*, we found two mRNA sequences (IDs: AY814744 and AY808628) with similarity to AGO1, three mRNA sequences (IDs: FN330861, AY809895, and AY809756) with similarity to AGO2, one mRNA sequence (ID: Sjc_0103990) with similarity to AGO3, and one protein sequence (ID: sjp_0117000) with similarity to AGO4. Therefore, we suggest that *S. japonicum *contains four members of the Argonaute protein family. These genes were named SjAGO1, SjAGO2, SjAGO3, SjAGO4, and SjDicer, respectively; however, all of them are partial coding sequences but SjAGO3. The complete CDS of SjAGO3 gene is 2715 bp coding a 904 aa protein in size. Therefore, we further obtained the remaining sequences of these genes through experimental approaches.

### Exploring the entire coding sequences of SjDicer in the genome

Homologous analysis confirmed that the SmDicer gene is highly homologous to CCON0000000957. Therefore, we designed PCR primers(Table 1: primer1, 2 and 3 ) according to conserved regions of AY810618 and CCON0000000957 in order to amplify CDS of the SjDicer gene using parasite cDNA as template. Consequently, the majority of the SjDicer coding sequence was determined in this manner. Furthermore, the 5' and 3' ends of the SjDicer gene were cloned by RACE using the Full RACE kit (Table 1: primer 4 and 5). After sequence and concatenation, the whole coding sequence of SjDicer was obtained. We annotated the SjDicer gene according to known genomic and transcriptome data.

**Table 1:** Oligonucleotides used to characterize SjDicer and SjAGO1/2

Names			Sequences(5'-3')	The length of PCR productions
**SjDicer**	Primer 1	**Forward**	**ACTGGACGCTGCCTACGAAAGAA**	3512 bp
		**Reverse**	**TGGACTGATGATTGGGTTTTGGT**	
	Primer 2	**Forward**	**GTCACCAAAACCCAATCATCAG**	884 bp
		**Reverse**	**GACGGTGATTATGTTGCTTGT**	
	Primer 3	**Forward**	**TGCTGTCGTGACTCCGGGTTATC**	3406 bp
		**Reverse**	**TGGTGATTTTGGGATACAAGCAG**	
	Primer 4	**5'RACE outer**	**GGCAGCGTCCAGTAGGTCTAG**	111 bp
		**5'RACE inner**	**GGGGGTGGAAGGAAGTGGTC**	83 bp
	Primer 5	**3'RACE outer**	**TATTCGAATCTTTAGCCGGTGC**	573 bp
		**3'RACE inner**	**GAGTGTGAATCTAAATGGCAAGCA**	343 bp
	Primer 6	**Real time F**	**CGACCTCGACGAATGGTTGATG**	167 bp
		**Real time R**	**GTTGATGCTGTTCGAGATGGTTG**	
**SjAGO1**	Primer 7	**Forward**	**CCAGGACGAGGTTCAGAAGGT**	516 bp
		**Reverse**	**CCATACTTCTCGACCACCGCC**	
	Primer 8	**Forward**	**GGCTCGTTCAGCTCCTGATCG**	920 bp
		**Reverse**	**GACCTTCACCAACACCATCTC**	
	Primer 9	**5'RACE outer**	**CCTGAAGATCCGCAGCCGACT**	326 bp
		**5'RACE inner**	**TTACGGTTCCAGAGACGGCGT**	235 bp
	Primer 10	**Real time F**	**GAATTCAGGGTACGTCCAAAC**	145 bp
		**Real time R**	**GTTGGTGCTGGATAAGAAACG**	
**SjAGO2**	Primer 11	**Forward**	**GTTCATTGATTCAGCAGATCG**	490 bp
		**Reverse**	**TCATACATGACGTCTGCTAGG**	
	Primer 12	**5'RACE outer**	**ACTTGGAGGTGGCCCATCTATG**	228 bp
		**5'RACE inner**	**GACGGTCTATACGAGCTACGGG**	173 bp
**SjGAPDH**	Primer 13	**Real time F**	**CAGGGCTGCTTTTAACTCTGGTAA**	101 bp
		**Real time R**	**GGGTGGAATCATATTGGAACATGT**	

**Figure 1:**
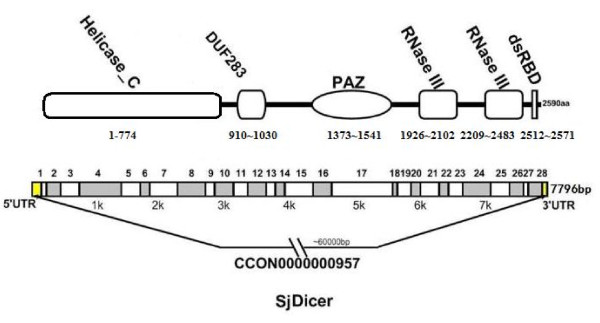
**Diagrammatic representation of SjDicer.** The SjDicer gene is approximately 60,000 bp in length and located on contig CCON0000000957. The complete mRNA of the SjDicer gene is 7,796 nt and contains 28 exons, which encode a 2590 aa protein, including an amino-terminal helicase domain, a DUF283(Domain of unknown function), a PAZ(Piwi-Argonaut-Zwille) domain, two tandom RNAse III domains, and an dsRBD(double strand RNA-binding domain).

A diagrammatic representation of the SjDicer gene is shown in Fig. 1. The size of the gene is approximately 60,000 bp. Its precise size is unknown, however, because the entire sequence of two introns is not complete, in spite of attempts to span the introns using PCR. The SjDicer gene includes 28 exons, ranging in size from 46 bp to 921 bp, forming a 7796 bp ORF. The SjDicer gene encodes a 2590-aa protein with a predicted molecular weight of 296,058 Da. Genome transcriptome analysis suggests that *S. japonicum *possesses a single Dicer gene. Alignment analysis revealed 71.8% identity between SmDicer and SjDicer proteins, and 9.2% identity among Dicers derived from *C. elegans*, *D. melanogaster*, *Aedes aegypti *and *H. sapiens*. Phylogenetic relationship analysis further demonstrated that SjDicer is an ortholog of the Dicer-like group and closely related to *C. elegans *and *S. mansoni *orthologs.

### Analysis of conserved protein domains in SjDicer

The BLASTp algorithm, underpinned by the Pfam (v22.0) and CDD databases, was used for searches of conserved protein domains or motifs from SjDicer sequences. Results revealed that SjDicer contains an amino-terminal helicase domain, a DUF283 domain, a PAZ domain, two tandom RNAse III domains, and a dsRNA-binding domain (Fig. 1).

Sequence analysis of SjDicer results revealed that the amino-terminal helicase domain possesses a conserved motif (^158^DECH^161^), indicating that it belongs to the DEAD-box protein family of RNA helicases[28]. The DUF283 domain of SjDicer proteins has a double-stranded RNA-binding fold. The PAZ domain of SjDicer is larger than its counterpart from other orthologs. The SjDicer protein contains a tandom RNase III domain. Table 2 shows that the tandem RNase III domain has four catalytic residues, ED and DE, which are important for function of the RNase III domain[14,20]. The dsRNA-binding domain exists at the carboxyl terminal of the SjDicer protein.

**Table 2:** Signature motif in the RNAse III and four catalytic residues of SjDicer protein

Dicer 1	**RNAse III**_**1**_	**RNAse III**_**2**_
SjDicer	ERL**E**TIG**D**......**D**CV**E**	QRL**E**FLG**D**.....**D**IF**E**
SmDicer 1	ERM**E**TIG**D**......**D**CV**E**	QRL**E**FLG**D**.....**D**IF**E**
CeDicer 1	ERF**E**TIG**D**......**D**AV**E**	QRL**E**FLG**D**.....**D**IF**E**
DmDicer 1	ERL**E**TIG**D**......**D**CV**E**	QRL**E**FLG**D**.....**D**VF**E**
AeDicer 1	ERL**E**TIG**D**......**D**CV**E**	QRL**E**FLG**D**.....**D**VF**E**
HsDicer 1	ERL**E**MLG**D**......**D**CV**E**	QRL**E**FLG**D**.....**D**IF**E**

### Exploring the entire coding sequences of SjAGO1 and SjAGO2 in the genome

AGO1 and AGO2 play a pivotal role in small RNA regulatory pathways (SRRPs)[6]. Therefore, our study focused mainly on SjAGO1 and SjAGO2. Transcriptome analysis of *S. japonicum *demonstrated that two ESTs, AY814744 and AY808628, were highly similar to the upstream and downstream regions of the SmAGO1 gene. At the same time, these analyses easily identified the 3' end of SjAGO1, since the extreme 3' end of SmAGO1 matched the sequence of AY814744. However, the extreme 5' end of the SjAGO1 cDNA is not well conserved and could not be identified by analysis of the genomic contig sequences. Moreover, there was no overlap between AY814744 and AY808628. Therefore, we designed primers based on the two known sequences, in order to amplify overlapping fragments of the predicted SjAGO1 coding gene. The 5' end of the SjAGO1 was cloned by RACE(Table 1: prmier 7, 8 and 9). Finally, the full SjAGO1 gene sequence was obtained after spliced sequences were concatenated. All exons combine to form a 3266 bp ORF, potentially encoding a 1009-aa protein with a predicted molecular weight of 111,647 Da.

**Figure 2:**
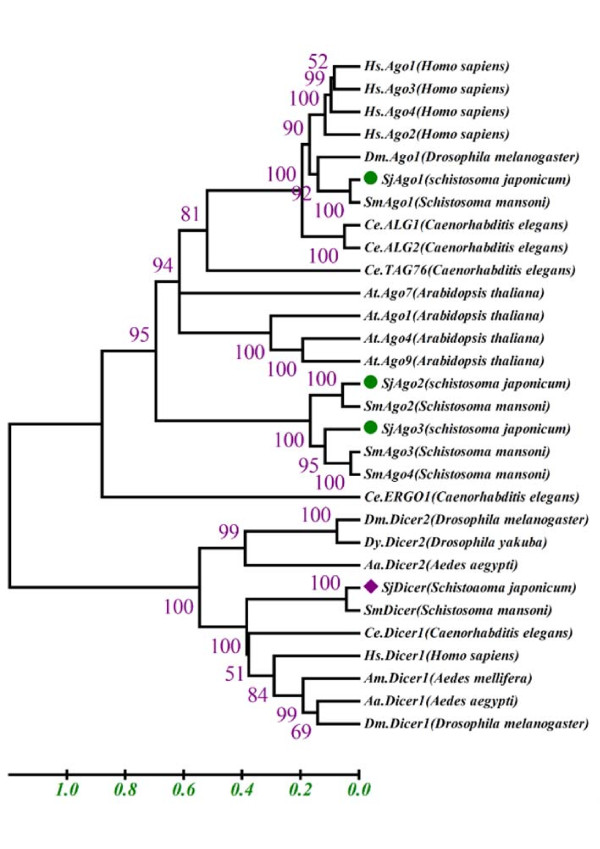
**Phylogenetic trees of Argonaute and Dicer proteins**. Multiple alignments were performed using ClustalX 2.0 and Mega 4.0 with bootstrap analysis. Bootstrap percentages are indicated at each branch. Phylogenetic tree of Argonaute proteins from plants, animals, and fungi. Accession numbers of sequences for Argonaute and Dicer proteins: Hs.AGO1 (NP_036331), Hs.AGO2 (NP_036286), Hs.AGO3 (NP_079128), Hs.AGO4 (NP_060099), Dm.AGO1 (NP_725341), Ce.ALG1 (NP_510322.2), Ce.ALG2 (NP_493837.1), Ce.TAG76 (NP_499192.1), At.AGO1 (gbAAB91987.1), At.AGO4 (NP_565633.1), At.AGO7 (NP_177103.1), At.AGO9 (CAD66636.1), Ce.ERGO1 (NP_503362.2), SmAGO1 (Smp_140010), SmAGO2 (Smp_179320), SmAGO3 (Smp_102690.2), SmAGO4 (Smp_102690.3), Dm.Dicer1 (NP_524453.1), Dm.Dicer2 (ABB54751.1), Dy.Dicer2 (ABB54764.1), Aa.Dicer1 (XP_001659747.1), Aa.Dicer2 (AAW48725.1), Hs.Dicer1 (NP_085124.2), Am.Dicer1 (XP_624510.2), Ce.DCR1 (NP_498761.1), SmDicer (smp_169750).

Regarding the SjAGO2 gene, three ESTs matched the sequence. Moreover, the 3' end of the SjAGO2 gene, including the stop codon and 3'UTR, were located on AY809756. Based on these sequences, we designed primers to amplify the unknown sequence by PCR. The 5' end was cloned by the 5' RACE(Table 1: primer 11 and 12). After sequence and concatenation, the whole coding sequence of SjAGO2 was obtained. All exons combine to form a 3199 bp open-reading frame, potentially encoding a 945-aa protein with a predicted molecular weight of 107,121 Da. The phylogenetic tree created with the Neighbor-Joining method also demonstrated that SjAGO1 and 2 are orthologs of the AGO-like group and closely related to *C. elegans *and *S. Mansoni *orthologs (Fig. 2).

### Analysis of conserved protein domains in SjAGO

**Figure 3:**
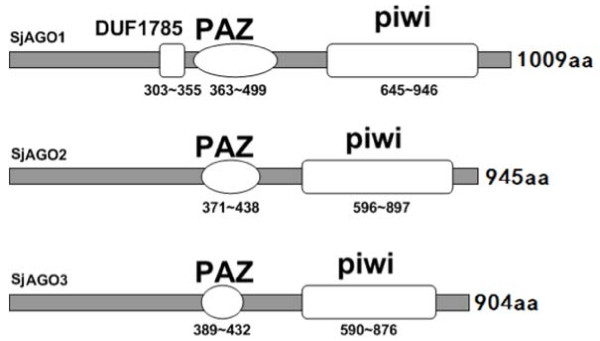
**Diagrammatic representation of the conserved domains of the SjAGO protein family.** They all contain PAZ and PIWI conserved domains. In addition, the SjAGO1 gene has a DUF1785 domain. SjAGO1, 2, and 3 proteins are 987 aa, 924 aa, and 904 aa in size, respectively

The alignment of AGO-like members revealed that most of their components, including SjAGO1/2, exhibited conservation of key amino acid (SjAGO1: D747/D819/H957 SjAGO2: D692/D774/H907) residues that coordinate Mg^2+ ^at the PIWI domain. Further, alignments between SjAGO1/2 proteins revealed 58% identity. Like all members of the AGO family in other species, SjAGO1/2 also have two main domains, PIWI and PAZ (Fig. 3). The core region of the PAZ domain contains 136 and 67 aa residues in SjAGO1 and 2, respectively. The PIWI domain contains about 300 aa residues in SjAGO1/2. In addition, there was a DUF1785 (domain of unknown function-1785) domain in SjAGO1, which included 52 aa residues, the function of which is not yet clear.

### Analysis of expression profiles of SjDicer and SjAGO in different stages of the parasite by Real-Time PCR

**Figure 4:**
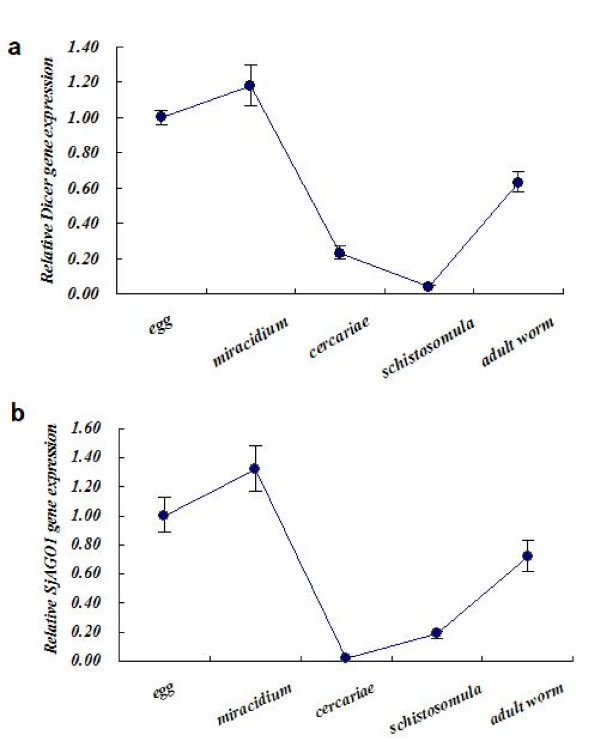
**Developmental expression analysis of *S. japonicum *(a) SjDicer and (b) SjAGO1**. The mRNA expression levels were measured, based on three replicates, in the life stages: eggs, miracidium, cercariae, schistosomula, and adult worms, using quantitative RT-PCR. Expression levels were calibrated according to the comparative 2-^ΔΔCt ^method, using the constitutively expressed SjGAPDH gene as an endogenous control, and were normalized relative to the egg stage.

We analyzed the expression profiles of SjDicer and SjAGO1 by relative qRT-PCR in different developmental stages of parasite, including eggs, miracidium, cercariae, schistosomula, and adult worms (Fig. 4). Detection was normalized to expression of the endogenous control SjGAPDH(Table 1: prmier 6, 12 and 13). The data are presented as the fold-change in gene expression normalized to an endogenous reference gene and relative to the levels found in the egg stage. As shown in Fig. 4, the expression of both SjDicer and SjAGO1 was detectable in all investigated stages of parasite. Moreover, the expression of SjDicer and SjAGO1 was highly consistent in each stage, but their expression levels were stage-specific. The highest expression levels of both SjDicer and SjAGO1 were seen in the miracidium stage. Interestingly, their expression reduced dramatically following penetration of the cercaria into its host, and then proceeded to increase for the remainder of the organism's lifespan within the mammalian host.

## Discussion

In *S. japonicum*, miRNAs have been identified by traditional approaches or high-throughput sequencing[22,23]. Meanwhile, endo-siRNAs were also found in *S. japonicum *via Solexa technology[23]. All of these imply the presence of constituents of the small RNA regulatory pathways in *S. japonicum*. Through a combination of phylogenetic tree, similarity between domains and mining of the *S. japonium *database we were able to show putative sequences with conserved domains of the Small RNA-mediated gene silencing pathways in the parasite. The results revealed that there was a single Dicer and four members of the AGO family in *S. japonicum*, named SjDicer and SjAGO1, 2, 3, and 4, respectively. However, all of these are partial coding sequences. Our study mainly focused on SjDicer and SjAGO1/2 in an effort to elucidate their full-length gene sequences through experimental approaches.

Our data indicate that the SjDicer gene comprises 28 exons that potentially encode a 2590 amino acid protein. Like SmDicer, SjDicer protein contains all domains that are characteristic of metazoan dicers including an amino terminal helicase domain, DUF283, a PAZ domain, two RNAse III domains and an RNA binding domain[20,21]. The identity between the SmDicer and SjDicer is as much as 71.8% in protein level. Among these domains, RNase III domains specifically cleave one strand of dsRNA. The first RNAse III domain is predicted to cut the RNA strand bearing a 3'-hydroxy group approximately 21 nucleotides from the end. In conjunction with the PAZ domain, the RNAse III domain is thought to be responsible for determining the distance from the terminus of the RNA to the cleavage site[29]. Dicer proteins are key components of the biogenesis of small RNA such as miRNA and siRNA. For example, Lee et al.[30] showed that Drosophila Dcr-1 mutants were defective in processing miRNA precursors, whereas Dcr-2 mutants had reduced siRNA levels and a complete RNAi defect in the eye.

Different Ago proteins have been found in several organisms, ranging from a single member in *Schizosaccharomyces pombe*, to more than twenty in *C. Elegans*[13,31]. Alignments between SjAGO1 and SmAGO1(SjAGO2 and SmAGO2) proteins revealed 81.1%(81.2%) identity. The role of Ago family in Drosophila and in *C. elegans *development is well documented for *D. melanogaster *and *C. elegans*[32]. It was observed that Ago1/2 are primary effector proteins responsible for the small RNA silencing pathway in fly and in *C. Elegans*. For example, Okamura et al.[33] showed that Drosophila AGO1 is essential for mature miRNA production, whereas AGO2 is responsible for siRNA-directed RNAi. The PAZ domain of Argonaute is a small RNA-binding domain, while the PIWI domain is an RNase-H-type domain that relies on divalent cation binding to facilitate dsRNA-guided cleavage of ssRNA [34].

Most small-RNA regulatory pathways are expressed in a developmental or organ-specific manner, or both, which provides information regarding their functions. As mentioned, SjDicer and SjAGO1 are crucial proteins in the miRNA regulatory pathway. Therefore, we analyzed the expression of SjDicer and SjAGO1 by relative qRT-PCR in different developmental stages of *S. japonicum*. The expression patterns of SjDicer and SjAGO1 during the life cycle of *S. japonicum *indicate that the miRNA regulatory pathway might take part in the transformation and development of *S. japonicum*.

## Conclusions

In summary, This study demonstrated existence of Dicer and Argonaute genes in *S. japonicum *and the 2590 amino acid residues of SjDicer containing 5 conserved domains, and the 1009, 945, and 904 amino acid residues of the SjAGO1, 2, and 3, respectively. In addition, expression profiles of SjDicer and SjAGO1 genes showed consistent expression of both genes in different stages, but difference in their expression levels. The study provides a theoretical platform for exploration of the functions of siRNA and miRNA in *S. japonicum*. Further investigations should be considered to explore the functions of the SjDicer and SjAGO families through experimental approaches.

## Competing interests

The authors declare that they have no competing interests.

## Authors' contributions

RL, XX and WP conceived and designed the experiments; RL, XX, ZW, YZ and JS performed the experiments and analysed the data. All authors read and approved the final manuscript.
